# Editorial: Functional hypothalamic amenorrhea seen from different perspectives

**DOI:** 10.3389/fendo.2023.1167668

**Published:** 2023-04-06

**Authors:** Emanuele Garzia, Anna Maria Marconi, Andrea Lania, Monica Rosa Miozzo, Elena Vegni, Alberto Priori

**Affiliations:** ^1^ Department of Mother and Child, Santi Paolo e Carlo Hospital, Milano, Italy; ^2^ Istituto di Medicina Aerospaziale “A. Mosso”, Aeronautica Militare, Milano, Italy; ^3^ Department of Health Science, University of Milano, Milano, Italy; ^4^ Department of Biomedical Sciences, Humanitas University, Rozzano, Italy; ^5^ Medical Genetics Unit, Santi Paolo e Carlo Hospital, Milano, Italy; ^6^ Unit of Clincal Psychology, Santi Paolo e Carlo Hospital, Milano, Italy; ^7^ Clinical Neurology Unit, Santi Paolo e Carlo Hospital, Milano, Italy

**Keywords:** amenorrhea, hypogonadotropic hypogonadism, osteoporosis, polycystic ovarian morphology, in vitro fertilization (IVF)

Functional hypothalamic amenorrhea (FHA) often represents a diagnostic and therapeutic challenge and affects the most diverse areas of medical clinics; to better understand its multifaceted characteristics, we tried to approach this disorder from different perspectives.

FHA is a form of Hypogonadotropic Hypogonadism (HH) in which anovulation and estrogen deficiency are expressed by inadequate endometrial growth, absence of menstruation, and infertility and can have lasting adverse effects on bone mineral density and trophism of genital and other target tissues (1). It can be related to psychological stress, decreased energy intake and strenuous exercise (2). FHA is considered “functional” because it can regress through correction or improvement of the behavioral factors that cause it.

Stress conditions play a primary role in promoting FHA. In the presence of similar stressors, interindividual variability in stress response results in inhibition of the HPG axis in some women and not in others. Recent studies, comprehensively reviewed by Fontana et al., have demonstrated a genetic contribution to FHA. Rare or polymorphic variants in genes controlling the development and/or function of GnRH neurons have been recognized in both idiopathic HH and FHA women, suggesting the presence of inherited susceptibility to functional impairment of GnRH secretion. Epigenetic changes have also been associated with different pathways involved in the HPG axis and, therefore, may participate in FHA and confer a personal predisposition to anovulation. Federici et al., in a large cohort of patients with congenital HH (CHH), identified gender differences in clinical presentation that could indicate variable expression of rare genetic variants.

The effect that long-term exposure to stress has on energy metabolism and reproduction is almost certainly caused by alterations in kisspeptin secretion from the arcuate nucleus of the hypothalamus, resulting in reduced GnRH drive. Although this effect was thought to be mediated by glucocorticoid receptors on kisspeptin neurons (3), Huang et al., using a special mouse model, have elegantly demonstrated the secondary role of these receptors and the possible involvement of receptors, including those for insulin, adiponectin, or leptin.

The diagnosis of FHA is based on clinical presentation and plasma hormone assays, both of which are characteristic (1). However, because some FHA patients with polycystic ovary morphology share features with a certain phenotype of women with polycystic ovary syndrome (PCOS), the differential diagnosis between these two disorders may not be easy. In fact, these are the two most frequent causes of secondary amenorrhea. Over the past two decades, some authors have proposed that these conditions may coexist (4, 5), but the majority support the clear distinction between them. Beitlt al. demonstrated that plasma hormone assays can be adequately sensitive to distinguish FHA women from PCOS women and proposed an original linear discriminant model using plasma assays of testosterone, SHBG, and gonadotropin.

One of the most important and potentially lasting consequences of FHA is bone loss secondary to metabolic and endocrine imbalances. Indirli et al. provided a thorough review on this issue, emphasizing the need for comprehensive clinical and instrumental evaluation, the utility of lifestyle interventions, and an optimal estrogen replacement strategy. Behary et al. reviewed the available evidence on several alternative and novel pharmacological interventions for the treatment of FHA-related bone loss, in addition to transdermal 17β-estradiol, which is currently the preferred intervention.

The low nutrient availability due to reduced dietary intake and/or high-energy exercise in women with FHA deserves to be rebalanced. The negative effects of hypoestrogenism on target tissues can be partially offset by hormone replacement treatment (HRT), which is more effective on uterine trophism than on skeletal homeostasis. For patients with fertility needs, ovarian stimulation with gonadotropins or pulsatile GnRH is used to induce ovulation. Pulsatile GnRH would be the most physiological-like method, but it requires close monitoring and its compliance is made difficult by the use of a portable pump injection device. In clinical practice, the most used ovulation induction procedure is daily gonadotropin injection, usually followed by oocyte retrieval, *in vitro* fertilization (IVF), and embryo transfer. Retrospectively evaluating the reproductive outcome of 81 patients with HH and 112 controls who underwent an IVF procedure, Zhang et al. showed that there were no significant differences between the two groups in IVF-related parameters and cycle outcomes. Since FHA patients are characterized by very low gonadotropin levels, it was hypothesized that in addition to stimulation with FSH, serum LH supplementation may be necessary to promote meiosis and the final stages of antral follicular growth (6). Di Segni et al. set out to evaluate the possible effect of exogenous LH supplementation on fertility-related outcomes in women with FHA. They concluded that available data demonstrate a positive effect on IVF cycle outcomes and support LH supplementation in women with FHA.

Although a psychogenic component has been recognized in FHA since its first diagnostic formulation (7), the role of psychological factors in the onset or persistence of this disorder is still poorly understood. Bonazza et al., analyzing published evidence, identified some recurrent psychological factors that could be clinically relevant, such as depression and dysfunctional attitudes toward eating, a higher level of perfectionism, greater concerns about errors, and a greater need for approval than controls. A tailored behavioral psychological intervention offers an effective treatment option that complements the clinical approach aimed at the recovery of ovarian activity.

Since heart rate variability (HRV) is a reliable measure of psychophysiological response to stress and coping to stimuli, Maiorana et al. examined changes in HRV during observation of erotic, neutral and disgusting images in a group of patients with FHA compared with controls. The results showed that patients with FHA had significantly higher HRV activation. This elevated HRV reactivity could reflect maladaptive activation of the parasympathetic nervous system and reduced reactivity with a delayed response to meet the environment demands.

A multidisciplinary approach is critical in the clinical management of FHA; it must involve a collaborative process among gynecologists, geneticists, endocrinologists, neurologists, clinical psychologists, and psychiatrists to facilitate proper diagnosis and provide the most appropriate treatment.

## Author contributions

EG and AM conceived the project; AL, AP, MM and EV created the prerequisites and encouraged the collection of contributions. All authors collaborated in the successful development of the Research Topic. All authors contributed to the article and approved the submitted version.
